# A Unique Case in Obstetrics

**Published:** 1866-02

**Authors:** E. Howard Irwin

**Affiliations:** Lodi, Wis.


### A UNIQUE CASE IN OBSTETRICS.

*Firm Attachment of Fœtal Head to Uterine Parietes.* By E. Howard Irwin, M. D.

On Sunday night, Oct. 2d, 1864, I was called to see Mrs. B——, a robust woman, aged 44, in labor with her fourth child. The case appeared quite natural, pains regular, and at about ten minutes interval. Upon examination per vaginam, the os uteri was found sufficiently dilated to admit the point of the finger, and the head could be detected at the superior strait by its volume and firmness. After watching her labor for about nine hours, I found though the pains had grown quite frequen and severe, that she had made but little progress, as the dilatation was but slightly increased. Attributing this state of things to rigidity of the os uteri, I placed her upon her feet, and bled her to approaching syncope. The relaxation not being as great as desired, she was put upon nauseating doses of tartar emetic, continued for about six hours, when the os uteri was dilated to about the diameter of a half dollar.

About fifteen hours had passed since my arrival, the labor had been quite severe, the membranes had ruptured, though *no waters* escaped, and an accurate examination had revealed that the anterior fontanelle was behind the symphysis pubis, and the posterior in front of the sacrum. In passing the finger within the circle of the os uteri, intending to increase its dilatation, I found that a portion of its circumference was soft cushion-like and quite dilatable, but that the finger was arrested in its progress around the head, at a point at the brim of the pelvis, a little to the left of the symphysis pubis, and could not there be passed between the child's head and the womb. The membranes could not be distinguished as an intervening structure, and it seemed as though the scalp, membranes and womb were united into a common structure, and that the left lateral surface of the os uteri was involved in the union. Under these circumstances, I called W. S. Schermerhorn, M. D., a skilful and experienced physician, who has practised by my side for the past ten years. After consultation, we concluded to continue the means to produce greater relaxation, hoping that in time nature would overcome the difficulties, so that the os uteri would become fully dilated, when if nothing but the irregularity of the head presentation remained, we would turn the forehead to one of the acetabula, that delivery might be accomplished by the natural powers.

The pains continued good for a few hours, but gradually lessened in frequency and force, so that we concluded to revive with ergot. She was accordingly put under its influence, and for a considerable length of time, the pains were of the most urgent character. Notwithstanding these powerful contractions, there was but little alteration in the condition of the os uteri, the dilatability was probably somewhat increased, but the actual dilatation was but slightly improved. The gradually lessening pains, within a short time had almost entirely ceased, and symptoms of exhaustion had appeared, indicated by great jactitation, wandering delirium, and an exceedingly rapid pulse. We feared that delivery had been procrastinated too long, and that death would shortly come to her relief. Restoratives were administered, and she was allowed a little rest, and comforted with the assurance, that as soon as sufficiently revived, we would deliver her instrumentally.

It was evident that immediate delivery must be secured to rescue the woman from impending death, and as the forceps was not adapted to the case, both from insufficient dilatation, and uncertainty as to exact nature and extent of the difficulties, delivery was undertaken by craniotomy, as the only means in our opinion that would save the life of the mother.

After allowing the woman a little rest, a most accurate examination was made, the os uteri was dilated about the diameter of an inch and a half, the vertex had sunk a little lower in the pubis, but the original difficulty still existed. The woman being conveniently placed, I perforated the head with the craniotomy scissors, making two incisions at right angles, each about an inch and a half in length, the brain was then freely broken up and was evacuated rapidly. I then introduced a pair of curved craniotomy forceps, and succeeded in getting a firm hold of the head. Dr. S. introduced the index fingers into the vagina to protect the structures of the mother from injury in the delivery of any sharp pieces of skull, and found, that when traction was made, the head descended somewhat, but that the womb descended also. It was then ascertained that the scalp of the child was so firmly attached to the womb of the mother, that it would require a considerable amount of well directed force to separate them. The tractions were continued, while Dr. S. separated the adhesions by a compound movement with the fingers, keeping the attached portion of the neck of the womb stationary during the process, and at the same time forced the head toward hollow of the sacrum. After about three hours increasing efforts the attachments were separated, the mouth of the womb then became fully dilated, and the head was delivered, the greater portion of the occipital bone having been received piecemeal. I now turned the face toward one of the groins, and brought the shoulders into the long diameter of the outlet; we now aroused the woman from the same unconscious condition in which she had lain during the operation, and solicited her efforts to bear down, but she was too feeble to render much assistance, and I delivered the child *very slowly*, giving the uterus time to contract as its cavity was emptied. Frictions over the uterine tumor were applied, stimulants and ergot given, from which we secured considerable contractions, and the placenta was delivered within an hour.

The adhesions were found to embracc that portion of uterus, lying internal to the descending ramus of the left os pubis, and involved the region of the right temporal ridge of the child's scalp. The internal surface of the neck of the womb was red and dry, and one or two ulcerated surfaces were exposed, a paste-like looking substance appeared to be the connecting medium, which was applied so closely to the hair and scalp, that it required to be literally peeled from the uterus, leaving the connecting medium, (which I suppose was the membranes,) attached to the scalp. I watched the patient very closely for about a week. She was well nursed, and, notwithstanding there was *no secretion of milk*, recovered without any untoward occurrence, except a copious crop of boils upon the external genitals.

Lodi, Wis., January, 1866.

